# Imaging features of invasion and preoperative and postoperative tumor burden in previously untreated glioblastoma: Correlation with survival

**DOI:** 10.4103/2152-7806.68336

**Published:** 2010-08-10

**Authors:** Patrick J. Kelly

**Affiliations:** 14 Sutton Place South, New York 10022, USA

The authors[1] have done a careful analysis of T1 gadolinium-enhancing volumes (T1) and T2 prolongation volumes in a series of patients harboring glioblastomas (GBMs). They found that the ratio of T2:T1 did not impact survival. In fact in many GBMs, it is a moot point anyway; resection of the T2 volume, thought to contain isolated tumor cells (ITCs), is usually the resection of “sick” but functioning, intact parenchyma with attendant neurological deficit.

In addition, they found that the spread and volume of the T1 abnormality to be a more robust indicator of prognosis. However, it is important to realize that the survival clock starts ticking when surgery is performed and the diagnosis is made. Patients with smaller tumors are most likely at an earlier stage of the disease and will, therefore, have a longer interval between diagnosis and death than those with larger tumors, in which the diagnosis has been made later in the course of the disease. And whether or not the extensiveness of surgery, beyond an internal decompression, has anything to do with actual significant prolongation of survival in these lesions is debatable, although I have always believed that it does.

We know that survival of a GBM patient with no therapy is about 17 weeks after diagnosis; with surgery and radiation, about 37 to 50 weeks; and somewhat longer with modern chemotherapy. But does surgery do much more than an internal decompression — and nowadays that's resection of the contrast-enhancing mass — or are we really providing a significant reduction of tumor burden that impacts a significant prolongation of survival?

Many published studies of surgical series over the past 50 years have attempted to show the benefit or lack of benefit of aggressive surgery in the survival of patients with glioblastomas. The problem is that GBM patients comprise a heterogeneous group. Many non-therapeutic variables impact the natural history of the disease over and above any therapy that these patients have received. The grade of tumor malignancy, age at diagnosis and performance status have much more influence on the length of survival following diagnosis than does the extent of surgery. In fact, in some series, extent of resection doesn't even make it onto the radar screen of statistical significance. There are several confounding factors.

By strict definition, glioblastoma multiforme is supposed to be a Grade IV astrocytoma. However, some neuropathologists include malignant glial tumors that have not only tumoral astrocytes but also tumors that exhibit necrosis and additional cell phenotypes: oligodendroglial and even primitive neuronal components. These mixed malignant tumors have been shown to have a slightly better prognosis than true glioblastomas that comprise only an astrocytic phenotype.

Probably the most significant problem with studies such as this is surgical selection bias: experienced surgeons know which patients will do well with extensive surgery and which will most likely do poorly. They select their cases accordingly. Good candidates: younger patients with smaller superficial, surgically accessible lesions confined to one hemisphere and usually sited in nonessential brain tissue who will do well with surgery — are selected for extensive resection. Elderly, neurologically-impaired patients with deep-seated, bi-hemispheric and/ or radiologically diffuse lesions will undergo biopsy or partial resection. It is, therefore, not surprising that in the present series that there was no difference in survival of patients undergoing subtotal resection or biopsy. It is also not surprising that the patients who underwent resection did better; they were better surgical candidates, to begin with.

My own experience supports the authors' observation that patients with greater midline shift had prolonged survival; in these tumors, the solid tumor tissue component (the contrast- enhancing mass; i.e., T1) grows as a solid mass that displaces rather than destroys and replaces brain tissue. Other GBM patients can have a 4 centimeter diameter contrast-enhancing tumor with little or no mass effect. In these cases, the tumor has *replaced* rather than displaced the brain tissue. One would expect that internal decompression provided by surgery would provide more benefit in patients with the lesions that displace brain tissue rather than in lesions that invade and replace brain tissue.

That the ratio of T1 gadolinium (Gd) contrast enhancement to T2 has no association with outcome is also not surprising. Neither T1 Gd enhancement nor T2 prolongation shows tumor! These imaging characteristics are epiphenomena of tumor being there. Gd enhancement is due to neovascularity and the inability of tumor blood vessels to maintain a blood-brain barrier. True, tumor tissue in malignant gliomas (and some nonmalignant gliomas; e.g., pilocytic astrocytomas, gangliogliomas, etc.) does induce neovascularity. But sometimes it does not, at least not to the degree to exhibit contrast enhancement. Similarly, T2 prolongation (“edema”) does not necessarily indicate invasion by isolated tumor cells, *per se*. Increase in the extracellular fluid may be due to the metabolic by-products of an increased number of cells in the microenvironment of the neuropil but could also be due to cytotoxic effects. And in serial stereotactic biopsy studies, we have found tumor cells far afield of any MRI-defined abnormality. However, the biology and mitotic potential of these cells probably determine a tumor's behavior and not necessarily the actual number of cells.

I agree that the existence and extension of ITCs beyond resectable limits are the reason that we as surgeons, have never been able to cure these lesions. We couldn't cure them 50 years ago, when surgical technology was relatively primitive; we can't cure them now in spite of all the expensive surgical technology that we have thrown at this problem over the past few decades. And unless our basic paradigm changes, we will never cure malignant gliomas. The fact is that we do not understand the biology of these lesions and we are making the diagnosis far too late in the course of the disease to make any significant difference in its natural history. By the time glioma patients exhibit symptoms, the vast majority of them are incurable. With treatment, it's just a matter of time until the disease kills the patient.
